# Metabolic modeling and analysis of the metabolic switch in *Streptomyces coelicolor*

**DOI:** 10.1186/1471-2164-11-202

**Published:** 2010-03-26

**Authors:** Mohammad T Alam, Maria E Merlo, The STREAM Consortium, David A Hodgson, Elizabeth MH Wellington, Eriko Takano, Rainer Breitling

**Affiliations:** 1 Groningen Bioinformatics Center, Groningen Biomolecular Sciences and Biotechnology Institute, University of Groningen, Kerklaan 30, 9751 NN Haren, The Netherlands; 2 Department of Microbial Physiology, Groningen Biomolecular Sciences and Biotechnology Institute, University of Groningen, Kerklaan 30, 9751 NN Haren, The Netherlands; 3 Department of Biological Sciences, University of Warwick, Gibbet Hill Road, Coventry CV4 7AL, UK; 4 Integrative and Systems Biology, Faculty of Biomedical and Life Sciences, University of Glasgow, Glasgow G12 8QQ, UK

## Abstract

**Background:**

The transition from exponential to stationary phase in *Streptomyces coelicolor *is accompanied by a major metabolic switch and results in a strong activation of secondary metabolism. Here we have explored the underlying reorganization of the metabolome by combining computational predictions based on constraint-based modeling and detailed transcriptomics time course observations.

**Results:**

We reconstructed the stoichiometric matrix of *S. coelicolor*, including the major antibiotic biosynthesis pathways, and performed flux balance analysis to predict flux changes that occur when the cell switches from biomass to antibiotic production. We defined the model input based on observed fermenter culture data and used a dynamically varying objective function to represent the metabolic switch. The predicted fluxes of many genes show highly significant correlation to the time series of the corresponding gene expression data. Individual mispredictions identify novel links between antibiotic production and primary metabolism.

**Conclusion:**

Our results show the usefulness of constraint-based modeling for providing a detailed interpretation of time course gene expression data.

## Background

The transition from exponential growth to stationary phase is a major event in microbial physiology [1]. During the exponential phase of growth, bacterial cells produce metabolites necessary for growth and grow rapidly. Once essential nutrients have been depleted, cells switch to stationary phase, stop growing, reorganize their energy metabolism and often start producing a new set of secondary metabolites, including antibiotics [2].

In this study, we have explored the metabolic switch in *Streptomyces coelicolor*, the model organism of the antibiotics producing genus *Streptomyces*. The genome of this soil bacterium has been sequenced and contains about 7825 genes, one of the largest numbers for any bacterium [3]. More than 20 clusters coding for the 4 known and several predicted antibiotics or related compounds have been identified in the genome [4]. To optimize the production of valuable secondary metabolites, understanding the shift from primary to secondary metabolism during the transition phase will play a key role.

We constructed a constraints-based genome-scale stoichiometric model of *S. coelicolor *metabolism, based on earlier similar models [5,6], and integrated the model predictions with a large gene expression dataset [7]. The constraints-based approach, in particular flux balance analysis, has been shown to be highly predictive of growth phenotypes in many microbial systems [8,9] and can be used to construct large scale metabolic models based on genome sequences in the absence of kinetic information, making it particularly attractive for less well-studied organisms like *S. coelicolor*.

Predictions from constraint-based models usually hold for steady-state assumptions [10,11]. To enable the incorporation of experimental information from time-series measurements, we extend the approach by applying a dynamically changing input function (specifying constrains on nutrient uptake) and objective function (specifying the shift of cellular resources from cellular growth to antibiotics production). The predicted flux profiles are then compared to the gene expression profiles of the corresponding enzyme-coding genes to validate the model.

We observe a surprisingly good correlation between predicted fluxes and measured gene expression, indicating both the power of the constraint-based modeling approach and the tight regulation of gene expression in *S. coelicolor*. A small number of incorrectly predicted fluxes indicate the need for including additional gene regulatory constraints to the model [12,13], but also allows the sensitive identification of misannotations and putative novel reactions involved in secondary metabolite biosynthesis.

## Results and Discussion

We have reconstructed a genome-scale model of *Streptomyces coelicolor *metabolism with recent updated annotations as discussed in the Methods section. Our aim was to study the metabolic switch between the primary phase and secondary phase of growth.

### Initial model validation

**Figure 1:**
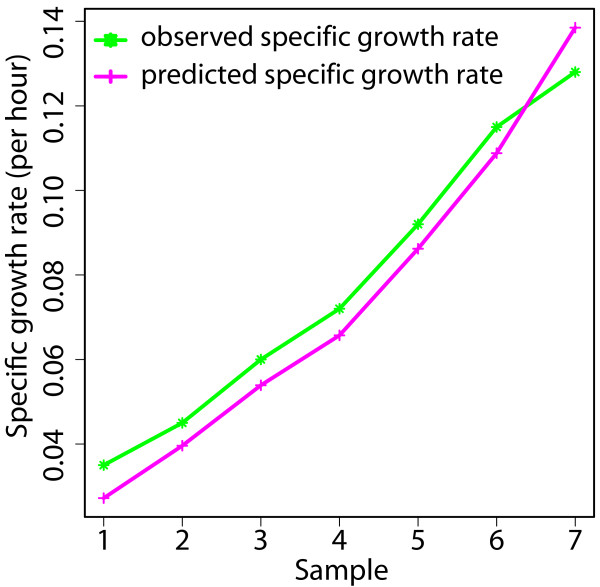
**Model validation**. Comparison of the experimentally observed specific growth rate from chemostat data [14] and the predicted *in silico *specific growth rate from the model in glucose limited media. The specific rate of glucose consumption, oxygen consumption, carbon dioxide production and actinorhodin production from 7 different conditions were taken from [14] and used as initial condition in the model.

To validate our model we first compared predicted growth rates to those reported for glucose limited environments [14]. In that work, *S. coelicolor *had been grown in chemostat culture in a chemically defined medium under various nutrient limitations. As the dilution rate of the chemostat is the same as the specific growth rate at steady state we can compare it directly to the prediction of the *in silico *model. In our model we used an input function that mimics the glucose limited medium used in these experiments, adopting the observed glucose and oxygen uptake rate as well as carbon dioxide and actinorhodin production rates as initial conditions in the model. We maximized biomass production to predict the optimized *in silico *specific growth rate and we compared the predicted growth with the observed growth. Figure 1 and Table 1 show that observation and prediction are in good agreement, indicating the general validity of our model.

**Table 1:** Comparison of experimentally observed dilution rates from chemostat data [14] and predicted specific growth rates

Glucose (mmol/g.h)	O_2 _(mmol/g.h)	CO_2 _(mmol/g.h)	Actinorhodin (μ g/g.h)	Observed dilution rate D (/h)	Predicted specific growth rate μ (/h)
0.5	1.8	1.9	2	0.035	0.0272

0.6	2	2	2	0.045	0.0396

0.8	2.4	2.5	415	0.06	0.0539

0.9	2.5	2.7	152	0.072	0.0657

1.1	3.1	3.1	60	0.092	0.0862

1.85	6.6	6.7	7	0.115	0.1088

2.1	7.2	7	5	0.128	0.1385

### Global metabolic switching from primary phase to secondary phase of growth

For a more detailed understanding of the metabolic transition phase, we then modeled flux changes happening during fermentation culture on phosphate limiting medium. For this growth condition we had earlier collected a detailed gene expression time series.

**Figure 2:**
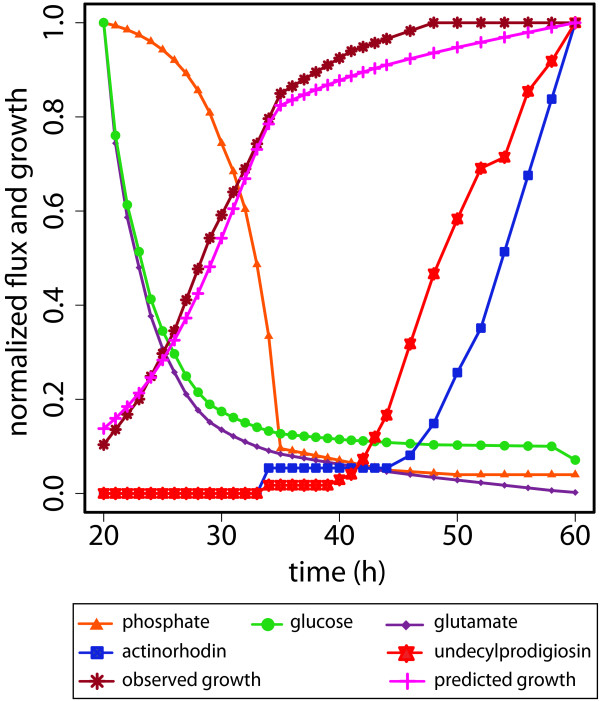
**Dynamic model constraints and predicted cell growth**. Based on online measurement on a fermenter experiment, normalized constraints of model influx of phosphate, glucose, and glutamate and the production of the antibiotics actinorhodin and undecylprodigiosin were determined. Their time course is shown together with the experimentally observed and *in silico *predicted growth.

Based on the measured nutrient uptake and product formation, we dynamically adapted the objective function and optimized the *in silico *specific growth rate. The optimum specific growth rate and optimal flux vector for all metabolic reactions were predicted for each time point. Figure 2 shows the observed normalized depletion of substrate glucose, glutamate and phosphate during growth. At about 34 hour, phosphate is depleted, triggering the transition to stationary phase and the production of antibiotics by the bacteria. The corresponding slow-down of growth matches well between prediction and observation.

**Figure 3:**
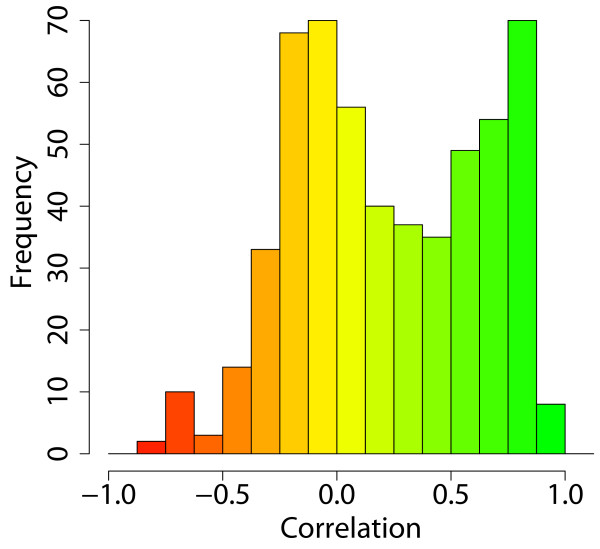
**Correlation between predicted flux and observed gene expression**. The histogram shows the correlation between gene expression and predicted flux for 549 enzyme-coding genes. A large number of enzyme-coding genes show high correlation. They include many primary metabolism genes and antibiotic biosynthesis genes. About half of the genes show poor correlation; these are mostly genes that show constant gene expression and/or predicted flux across the entire time course, leading to a correlation coefficient close to zero. A small but noteworthy number of genes show statistically significant negative correlation between gene expression levels and predicted flux. These cases are discussed in more detail in the main text.

Next we compared the predicted metabolic flux profile of all 549 enzyme-coding genes to the corresponding gene expression data from Nieselt et al. [7]. A histogram of correlation coefficients between predicted flux and observed gene expression is shown in Figure 3. The correlation of predicted flux and gene expression level is highly significant, and a large number of genes exhibit very high correlation (33% of genes; r > 0.5). This shows not only the global validity that these genes of our model are probably correctly annotated in the model but also illustrates the tight regulation of gene expression level for enzyme-coding genes of *S. coelicolor*. This is in agreement with the general observation that gene expression is more tightly regulated in unicellular compared to multicellular organisms, for evolutionary reasons, such as the much larger effective population size and stronger energetic constraints in small organisms [15,16].

A large set of genes does not show correlation (64% of genes; -0.5 < r < 0.5). These are mostly genes that do not change expression (nor predicted flux) along the time course. In these cases of constant expression no correlation information is present in the data, leading to correlation coefficient close to zero. Of course, there will also be cases where gene expression levels and flux levels do not correlate for other reasons, for instance due to post-transcriptional and post-translational regulation mechanisms.

**Figure 4:**
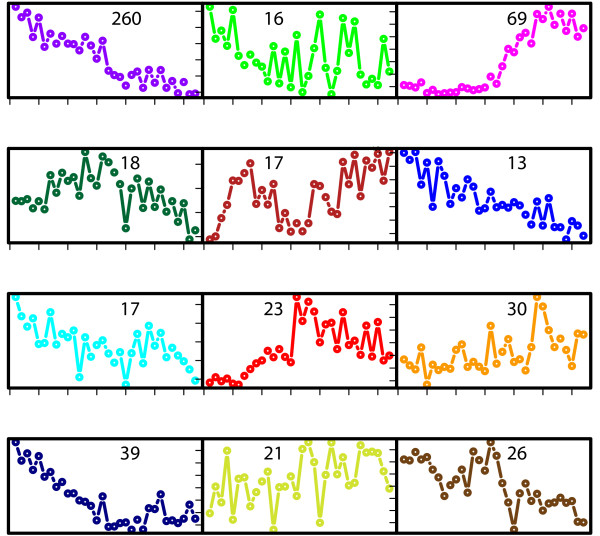
**Average expression profile of 12 expression clusters defined by hierarchical clustering**. Gene expression profiles of all enzyme-coding genes in our metabolic flux model were subjected in unsupervised clustering. The number of genes in each cluster is indicated. Several clusters show a clear expression trend matching the changing physiology of the fermentation. The pink cluster is the "antibiotics" cluster, switching on upon phosphate depletion; the purple cluster includes the majority of central metabolism genes that are down-regulated.

Strikingly, there is also a small group of strongly anticorrelating genes (15 genes; r < -.5). These are potentially the most interesting cases; they could indicate wrong annotations of gene function, but also the unexpected presence of regulatory constraints or novel functionalities of genes. To further examine these options, we subdivided all 549 observed expression profile into 12 clusters, based on unsupervised hierarchical clustering. The number 12 was chosen to allow sufficient resolution of different expression pattern. Figure 4 shows the average expression time course of each of the 12 resulting clusters. For instance, the pink and red clusters, which contain many genes involved in secondary metabolite production, switch on upon phosphate depletion. The purple, navy blue and blue clusters mostly include genes involved in central metabolism and anabolic functions and are down-regulated when nutrient resources in the medium are depleted.

**Figure 5:**
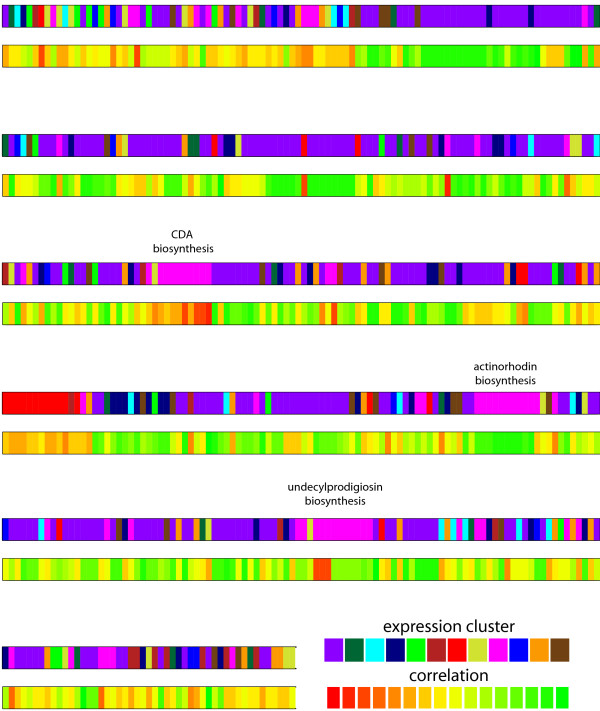
**Genome mapping of expression clusters and correlation between expression and predicted flux**. All enzyme-coding genes are shown arranged in their order along the chromosome. The upper trace colors genes according to their membership in one of 12 expression clusters (Figure 4); genes belonging to the same cluster tend to be neighbors along the chromosome, reflecting the operon structure of the genome. The lower trace shows how strongly the predicted flux for each gene correlates with its expression. Genes from some expression clusters tend to show good correlation to the predicted flux (green), e.g. those in the central metabolism cluster (purple); mispredictions (red) seem to cluster along the chromosome and normally affect genes that are upregulated in stationary phase (pink cluster). The position of three major antibiotics biosynthesis clusters is highlighted.

When mapping the gene expression clusters onto the genome (Figure 5) it is clear that genes with similar expression dynamics tend to be neighbors along the chromosome. Moreover, when also visualizing the correlation between gene expression and predicted flux, one can see the that strong anticorrelated expression is seen almost exclusively for genes in the pink and red clusters, which switched on expression during the transition phase, while the predicted flux for these genes are decreasing along the time course (Table 2). In contrast, many genes with high positive correlation belong to the purple, navy blue and blue clusters which contain genes of central metabolism, including biosynthesis clusters for arginine, cysteine, glutamate, glutamine, glycine, fatty acid, histidine, homoserine, isoleucine, leucine, lysine, methionine, N-acetyl muramic acid (NAM) and N-acetyl glucosamine (NAG), as well as sulphate metabolism. Expression of the antibiotic gene clusters for actinorhodin and undecylprodigiosin was also highly correlated to the predicted fluxes.

**Table 2:** List of anticorrelated genes

SCO ID	Definition	Pathway	r
SCO2286	alkaline phosphatase	folate biosynthesis	--0.80

SCO3249	[acyl-carrier-protein] S-malonyltransferase	fatty acid biosynthesis	--0.78

SCO5887	[acyl-carrier-protein] S-malonyltransferase	fatty acid biosynthesis	--0.75

SCO0386	asparagine synthetase (glutamine-hydrolysing)	aspartate metabolism	--0.75

SCO3248	pentadecanoyl-[acyl-carrier protein] synthesis	fatty acid biosynthesis	--0.74

SCO5886	pentadecanoyl-[acyl-carrier protein] synthesis	fatty acid biosynthesis	--0.71

SCO5888	pentadecanoyl-[acyl-carrier protein] synthesis	fatty acid biosynthesis	--0.70

SCO0828	alkaline phosphatase	folate biosynthesis	--0.67

SCO2068	alkaline phosphatase	folate biosynthesis	--0.66

SCO3246	pentadecanoyl-[acyl-carrier protein] synthesis	fatty acid biosynthesis	--0.63

SCO3595	D-alanine-D-alanine ligase	D-alanine metabolism	--0.63

SCO3221	prephenate dehydrogenase	tryptophan biosynthesis	--0.63

SCO6655	GTP cyclohydrolase II	riboflavin metabolism	--0.61

SCO6787	butyryl-CoA dehydrogenase	propanoate metabolism	--0.59

SCO2687	GTP cyclohydrolase II	riboflavin metabolism	--0.52

The most strongly anticorrelated genes are listed on the basis of correlation between gene expression and predicted flux (r < --0.25). Pathway annotations are based on the KEGG database without manual curation. Most of the genes belong to the pink or red cluster and are upregulated in stationary phase; these represent potential new genes involved in antibiotics biosynthesis. Several examples validating this interpretation are discussed in detail in the text.

One large group of anticorrelated genes is the set of 10 genes located the middle of the calcium dependent antibiotics (CDA) biosynthesis gene cluster (SCO3210-SCO3249) [17]. SCO3210 and SCO3221 are annotated as 2-dehydro-3-deoxyheptonate aldolase and prephenate dehydrogenase respectively, part of the shikimate pathway (tryptophan biosynthesis). Tryptophan is a precursor for CDA, and there are four anticorrelated genes (SCO3211-3214) which encode for enzymes TrpC2, TrpD2, TrpG, and TrpE2. It seems obvious that these genes are involved in the biosynthesis of tryptophan for CDA biosynthesis and not in the production of tryptophan for general primary metabolism. Indeed it has been shown that these genes do not complement a deficiency in central tryptophan biosynthesis [17]. SCO3249 encodes an ACP homolog, and the adjacent genes SCO3246 and SCO3248 along with SCO3228 are proposed to be involved in the biosynthesis of the *N*-terminal epoxyhexanoyl fatty acid side chain [17]. While the direct involvement in CDA biosynthesis has not yet been established for all of these genes, the non-complementation as well as the clear anticorrelation in our model analysis point to the existence of strong regulatory constraints on the expression of these genes. Such regulatory constraints are not routinely included in flux balance analysis, but can substantially enhance its predictive accuracy [12,13]. Our result shows that a lack of regulatory information can be efficiently compensated by the integration of transcriptomics information, which quite specifically highlights this group of genes for further study.

Another group of anticorrelating genes is seen in the middle of the undecylprodigiosin biosynthesis gene cluster [4,18]. Three genes (SCO5886, SCO5887 and SCO5888) in this cluster were automatically annotated in our model as fatty acid biosynthesis genes on the basis of sequence similarity with fatty acid genes (3-oxoacyl-[acyl-carrier-protein] synthase II, acyl carrier protein, 3-oxoacyl-[acyl carrier protein] synthase III). However these three genes are well known to be involved in undecylprodigiosin production under the gene names *redR *(SCO5886), *redQ *(SCO5887) and *redP *(SCO5888). This is a clear example of a misannotation that is revealed by the correlation analysis and can easily be fixed in the model.

A third example of strongly anticorrelated genes listed in Table 2 are three alkaline phosphatases - SCO2286 (*phoA*), SCO0828 (*phoC*) and SCO2068 (*phoD*) - which are assigned in the KEGG database (and consequently in our model) to the folate biosynthesis pathway. Their expression pattern, which shows strong induction upon phosphate depletion, is consistent with earlier reports on their control by PhoR/PhoP [19] and a potential role in secondary metabolism, but is less easy to reconcile with a putative function in folate biosynthesis, which is based only on sequence homology.

In all three of these cases, the integration of gene expression and model flux predictions highlighted groups of genes involved in antibiotics production. A small set of additional anticorrelated genes (Table 2) are widely scattered through out the genome (Figure 5). Each of them is a potential candidate from model correction and for the identification of new secondary metabolite biosynthesis genes with specifically constrained gene expression patterns.

Our biological understanding of *S. coelicolor *metabolism is further enhanced by a more detailed analysis of the reactions for which the flux balance analysis predicted zero flux. When clustering the measured gene expression profiles for the genes encoding the enzymes of these zero-flux reactions, a substantial number of genes showed consistent changes in gene expression along the time course, suggesting that the corresponding reactions are in fact active (Additional file 1). Striking examples include a large number of genes for vitamin B12 (cobalamin) biosynthesis, a group of ten genes involved in calcium-dependent antibiotic (CDA) biosynthesis, and three genes involved in ectoine biosynthesis (Additional files 2, 3, 4 and 5). Each of these cases provides important insights: the first one shows that vitamin B12 is likely to be produced by *S. coelicolor *under the growth conditions of our experiment, even if it is not essential due to the availability of cobalamine-independent enzymes [20]. The second one highlights that CDA biosynthesis genes are coherently induced in expression during the metabolic switch, similar to undecylprodigiosin and actinorhodin and concordant with the results of the correlation analysis discussed above. This could indicate that this additional antibiotic compound is potentially also produced in phosphate starvation conditions, contrary to previous expectations [21]. Finally, the case of ectoine biosynthesis genes suggests that this novel osmoprotectant metabolite is produced by *S. coelicolor*. This has in fact been experimentally confirmed recently [22]. In each of these cases, the activity of the pathway was not predicted, based on the biological evidence incorporated in the stoichiometric model and the expected biomass composition, and the comparison of flux balance predictions and gene expression data indicated relevant modifications of our metabolic model. A complete list of genes that have zero predicted flux but show gene expression is included in the supplementary material (Additional files 2, 3, 4 and 5).

Conversely, our model can be used to identify those genes that are predicted to be essential for growth (non-zero flux under all conditions), but show no or very low gene expression. There are 159 predicted essential genes in our model, which have a median log gene expression level of 7.47, compared to 6.83 for the non-essential genes and 4.66 for the negative controls. This indicates that on average the essential genes have a 60% higher expression than the non-essential genes. There is only one predicted essential gene with a detected median expression level below 5.0, compared to 23 non-essential genes with such low expression levels. This non-expressed essential gene is *panB *(SCO2256), a 3-methyl-2-oxobutanoate hydroxymethyltransferase of pantothenate and coenzyme A biosynthesis, which has a maximum log expression signal of only 5.53. Its apparent non-expression can be due to insufficient hybridization of the gene-specific probes on the microarray, but it could also indicate the existence of another isoenzyme or additional metabolic pathways that would make this reaction redundant. In both of these cases, this gene might warrant further detailed study.

The observed good correlation between gene expression and predicted metabolic flux is not necessarily expected; expression levels can show little correlation to protein levels, enzyme activity and metabolic flux for many reasons [23]. It could be that the relationship between expression and flux is tighter in prokaryotes like *S. coelicolor*, than in multicellular eukaryotic model organisms [15,16]. However, we cannot exclude that the group of non-correlated genes contains not only reactions with constant flux, but also reactions with dynamic flux little correlation between gene expression and protein activity or metabolic flux. In a next step, it will be interesting to directly incorporate the gene expression information in the model, providing additional constraints on the maximum flux [23,24].

## Conclusions

Our study demonstrates the ability of flux balance analysis to not only study classical steady-state conditions but also to predict microbial behaviour in dynamic growth conditions provided that sufficiently detailed measurements of the changing growth conditions (nutrient uptake) and cellular objective (antibiotic production rate) are available. In combination with detailed gene expression information, these dynamic model predictions can help identifying potential new players in the metabolic switch, including putative new genes for antibiotic synthesis.

## Methods

### Transcriptomics

The gene expression dataset used in this study has been described in detail in [7]. Briefly, *S. coelicolor *was cultivated in a phosphate limiting defined medium containing glucose as a carbon source and glutamate as a nitrogen as well as carbon source. Samples for transcriptomics and off-line analysis were taken every hour from 20 to 44 hours after inoculation (25 sample points), and subsequently every second hour from 46 to 60 hours after inoculation. Cell dry weight was measured on samples collected every third hour between 20 and 40 hours. The last sample, collected at the end of the fermentation (68 hours after inoculation), was used for analysis of remaining nutrients and total production levels of red and blue pigments. Only one sample was collected at each time point and no re-samplings were performed. Gene expression was measured on custom-made Affymetrix gene chips as described in [7]. Expression data have been deposited in the GEO database under accession number GSE18489. Measurements for all known or predicted enzyme-coding genes were extracted and matched to the corresponding reactions in the constraint-based model.

### Constraints-based genome-scale metabolic model reconstruction

A genome-scale stoichiometric metabolic model of *Streptomyces coelicolor *was reconstructed from different sources of data, including KEGG pathways, ScoCyc pathways, biochemistry textbooks, an extensive literature survey and available genome-scale models of other organisms. The initial stoichiometric matrix was generated based on KEGG and ScoCyc and manually curated to refine the *S. coelicolor*-specific parts of the metabolic network (e.g., antibiotic biosynthesis), to specify the correct reversibility constraints of reactions, and to add missing essential reactions. Missing essential reactions were identified iteratively; a minimum set of hypothetical reactions was added to the model if an essential metabolite could not be produced otherwise. Reversibility and essentiality of reactions were also compared to other published genome-scale models of *S. coelicolor *and other organisms [6,25-27]. The resulting model is very similar to the model of Borodina et al. [5,6], and differs mainly in the more comprehensive inclusion of antibiotic pathways.

In the final curated model, one lumped reaction is added to produce the biomass of the cell. Information about biomass composition and growth and non-growth associated ATP maintenance were taken from Borodina et al. [6] and Ingraham et al. [28] and complemented with literature information [29,30]. Some of the biomass precursor biosynthesis reactions are also lumped reactions, e.g. protein translation, and were specified according to the literature and published genome-scale models [6]. The full model in SBML format is available in the supplementary material (Additional file 2).

Analysis of the model was based on standard flux balance analysis (FBA) to predict optimal *in silico *growth and metabolic flux distribution using the COBRA tool [31]. Uptake fluxes for metabolites not available in the medium were set to zero, while metabolic by-products were always allowed to leave the metabolic system. Observed nutrient uptake rates from the fermenter culture used for the transcriptome analysis were used to define the constraints of nutrients uptake for the model (input function). The objective function was defined as maximizing the growth rate. Beginning at 34 hours, we dynamically varied the biomass composition by adding increasing amounts of antibiotics, based on the observed antibiotics production rate.

### Comparing transcriptome data and predicted flux

Our computational model contains 643 metabolites and 1015 reactions: 747 reactions for metabolite biosynthesis and degradation, 152 transport reactions, and 116 additional input and output constraints of the system. 666 reactions were annotated as enzyme-catalyzed reactions and could be matched to an enzyme-coding gene. Some reactions were annotated as potentially catalyzed by more than one gene and some genes catalyze more than one reaction. If one gene catalyzes multiple reactions, we matched its expression profile to the reaction with the maximum predicted flux, hypothesizing that this reaction will dominate the expression behavior. In total, 789 genes are assigned to 666 enzymatic reactions. Of these, 558 genes are predicted to have non-zero flux (the remaining 231 genes are not used for biomass production according to the model). Out of these 558 genes, 9 genes were involved in cell maintenance with constant flux and zero standard deviation; these were excluded from the further analysis. In total we therefore considered 549 enzyme-coding genes with non-zero predicted flux. For each of these genes, we compared the predicted flux profile and the observed gene expression levels using Pearson's correlation, testing whether gene expression was indeed upregulated when a much higher flux through a particular reaction was required at a certain growth phase.

## Authors' contributions

EMHW, ET and RB designed and coordinated the study. MTA carried out the modelling and drafted the manuscript. The STREAM consortium provided the expression data prior to publication. MTA and MEM integrated the model and expression data. DAH, ET and RB interpreted the results. EMHW, ET and RB revised the manuscript. All authors read and approved the final manuscript.

## Supplementary Material

Additional file 1 **Expression clustering plots**. PDF file depicting the expression clustering of 231 enzyme-coding genes for which the catalyzed reaction had zero predicted flux at all time points of the flux balance analysis of our model. The majority of genes are members of clusters that show highly consistent dynamics across the time course, e.g. the purple, red and pink clusters, indicating that they are indeed expressed and the corresponding reactions likely to be active. Click here for file

Additional file 2 **Stoichiometric metabolic model**. SBML file describing the metabolic model of *Streptomyces coelicolor*. Click here for file

Additional file 3 **Table of metabolites**. Excel table defining all metabolites used in the metabolic model. Click here for file

Additional file 4 **Table of reactions**. Excel table defining all reactions used in the metabolic model. Click here for file

Additional file 5 **Table of zero-flux reactions**. Excel table of reactions that show consistent zero predicted flux, including their membership in the expression clusters depicted in Additional file 1. Click here for file
