# Supplementary figures and images for: Metabolic modeling and analysis of the metabolic switch in Streptomyces coelicolor
Source: BMC Genomics. 2010 Mar 26;11:202. doi: 10.1186/1471-2164-11-202 (PMC2853524; doi:10.1186/1471-2164-11-202)

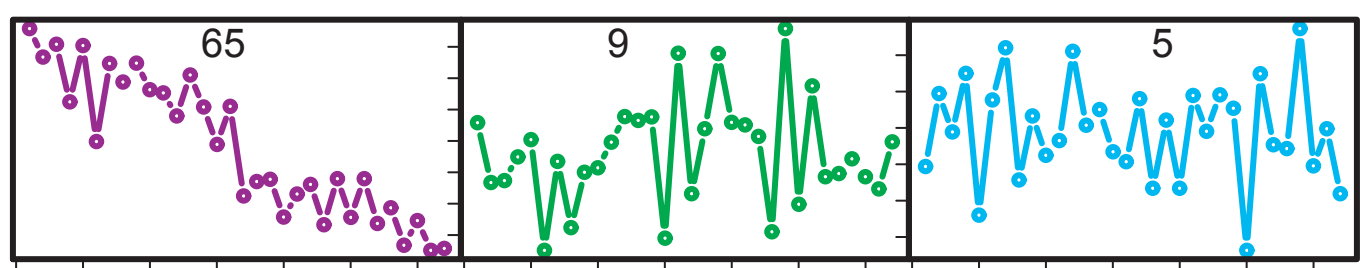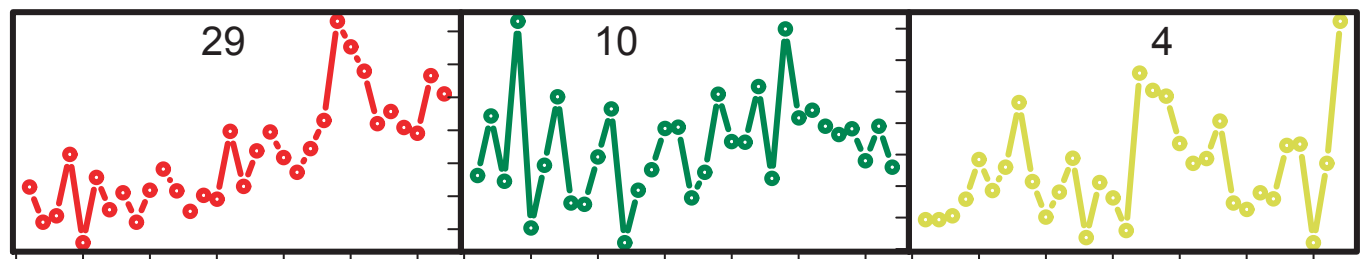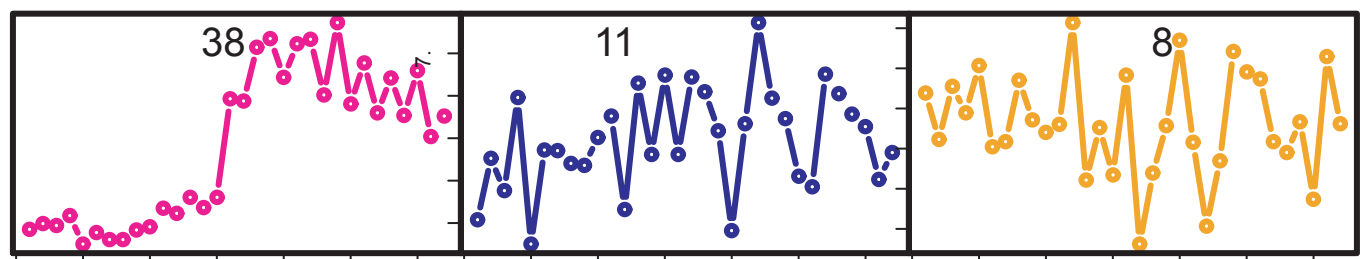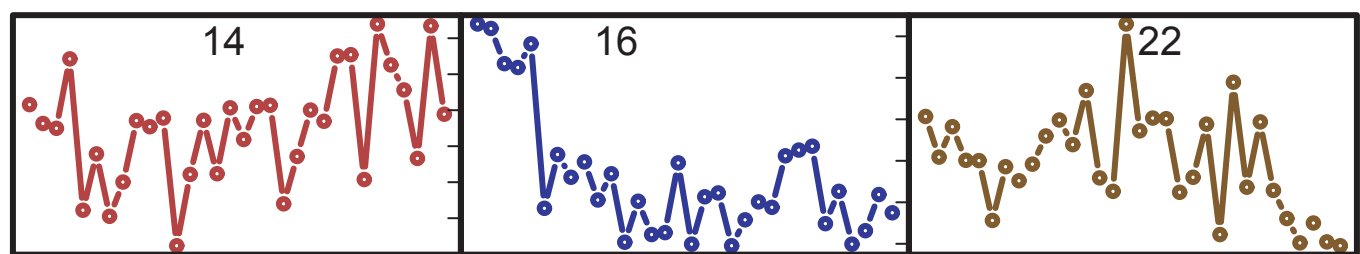

Supplement: Additional file 1 — Expression clustering plots. PDF file depicting the expression clustering of 231 enzyme-coding genes for which the catalyzed reaction had zero predicted flux at all time points of the flux balance analysis of our model. The majority of genes are members of clusters that show highly consistent dynamics across the time course, e.g. the purple, red and pink clusters, indicating that they are indeed expressed and the corresponding reactions likely to be active. [file 1471-2164-11-202-S1.PDF]
